# Synthetically enhanced: unveiling synthetic data's potential in medical imaging research

**DOI:** 10.1016/j.ebiom.2024.105174

**Published:** 2024-05-30

**Authors:** Bardia Khosravi, Frank Li, Theo Dapamede, Pouria Rouzrokh, Cooper U. Gamble, Hari M. Trivedi, Cody C. Wyles, Andrew B. Sellergren, Saptarshi Purkayastha, Bradley J. Erickson, Judy W. Gichoya

**Affiliations:** a Department of Radiology, Mayo Clinic, Rochester, MN, USA; b Department of Orthopedic Surgery, Mayo Clinic, Rochester, MN, USA; c Department of Radiology, Emory University, Atlanta, GA, USA; d Google Health, Google, Palo Alto, CA, USA; e School of Informatics and Computing, Indiana University–Purdue University, Indianapolis, IN, USA

**Keywords:** Synthetic data, Diffusion model, Generative AI, Data supplementation, Chest radiographs

## Abstract

**Background:**

Chest X-rays (CXR) are essential for diagnosing a variety of conditions, but when used on new populations, model generalizability issues limit their efficacy. Generative AI, particularly denoising diffusion probabilistic models (DDPMs), offers a promising approach to generating synthetic images, enhancing dataset diversity. This study investigates the impact of synthetic data supplementation on the performance and generalizability of medical imaging research.

**Methods:**

The study employed DDPMs to create synthetic CXRs conditioned on demographic and pathological characteristics from the CheXpert dataset. These synthetic images were used to supplement training datasets for pathology classifiers, with the aim of improving their performance. The evaluation involved three datasets (CheXpert, MIMIC-CXR, and Emory Chest X-ray) and various experiments, including supplementing real data with synthetic data, training with purely synthetic data, and mixing synthetic data with external datasets. Performance was assessed using the area under the receiver operating curve (AUROC).

**Findings:**

Adding synthetic data to real datasets resulted in a notable increase in AUROC values (up to 0.02 in internal and external test sets with 1000% supplementation, p-value <0.01 in all instances). When classifiers were trained exclusively on synthetic data, they achieved performance levels comparable to those trained on real data with 200%–300% data supplementation. The combination of real and synthetic data from different sources demonstrated enhanced model generalizability, increasing model AUROC from 0.76 to 0.80 on the internal test set (p-value <0.01).

**Interpretation:**

Synthetic data supplementation significantly improves the performance and generalizability of pathology classifiers in medical imaging.

**Funding:**

Dr. Gichoya is a 2022 10.13039/100000867Robert Wood Johnson Foundation Harold Amos Medical Faculty Development Program and declares support from 10.13039/100006098RSNA Health Disparities grant (#EIHD2204), Lacuna Fund (#67), 10.13039/100000936Gordon and Betty Moore Foundation, 10.13039/100000002NIH (NIBIB) MIDRC grant under contracts 75N92020C00008 and 75N92020C00021, and 10.13039/100000050NHLBI Award Number R01HL167811.

**Research in context**

**Evidence before this study**

Before undertaking this study, we conducted a comprehensive literature review using PubMed and Google Scholar databases, including pre-prints, to ensure a broad and current understanding of the field. The search terms used were combinations of “diffusion models”, “generative adversarial networks”, “GAN”, “variational autoencoders”, “VAE”, “synthetic∗”, and “medical imaging”. The time frame for the search was restricted from January 2015 to September 2023. We included studies published in any language that focused on the use of generative models, particularly GANs, VAEs, and diffusion models, in medical imaging. This encompassed original research, commentaries, and opinion articles. Studies were excluded if they were not in English, did not pertain to medical imaging, or fell outside the specified date range.

Previous studies have used Generative Adversarial Networks (GANs) and more recently, Denoising Diffusion Probabilistic Models (DDPMs), for creating realistic medical images. It has also been shown that using synthetic data can help train deep learning models that have improved performance on the same dataset. However, these studies did not clearly delineate the underlying causes for improved performance, such as possible leakage of distribution characteristics, increased dataset size, or disentangled distribution. To our knowledge, no meta-analysis or systematic review specifically addressed the use of these advanced generative models in the context of medical image synthesis. The evidence we gathered highlighted a growing interest in generative models for medical imaging but revealed a gap in comprehensive research specifically focused on the practical applications and performance implications of these technologies in healthcare contexts.

**Added value of this study**

This study adds significant value to the existing body of research by providing a focused analysis of synthetic medical images, specifically chest X-rays, and their implications on model performance and generalizability. Our research contributes to a deeper understanding of how DDPMs can be used effectively for dataset augmentation in medical imaging, offering insights into their potential to enhance the performance and generalizability of diagnostic models. We designed a comprehensive set of experiments to investigate the effect of synthetic data on downstream classifier performance. We showed that by iteratively adding synthetic data, model performance increased across different datasets. Additionally, using a synthetic dataset with two to three times the size of real data, the model's performance is comparable to that of a model trained only on real data, highlighting the potential for synthetic data sharing. Additionally, by mixing synthetic data with an external data source, we showed that the model generalization gap closes, providing promising avenues for clinical use cases.

**Implications of all the available evidence**

The implications of this study, combined with existing evidence, suggest that the integration of advanced generative models and synthetic images could be a pivotal step in overcoming limitations in medical imaging datasets, such as small volume. This approach has the potential to improve diagnostic accuracy and patient outcomes by enabling the development of more robust and generalizable diagnostic tools. Our findings also highlight the need for further research into optimizing these models and understanding their long-term implications in clinical settings, paving the way for more personalised and effective healthcare solutions.

## Introduction

Chest X-rays (CXR) are widely used as the primary imaging modality for a variety of conditions, ranging from acute respiratory distress to chronic pathologies like lung cancer. They are essential for quick and efficient patient triage, especially in emergency settings, and are the most frequently carried out diagnostic imaging exam.^1^ Several CXR triage tools have been approved by the FDA to detect pathologies like pneumothorax, pleural effusion, and rib fractures.^2^ Like other DL-based models, the proposed solutions are not without their shortcomings; most importantly, these models do not always generalise and can suffer from decreased performance when applied to new populations.^3^ For instance, a recent study retrospectively evaluating four commercial tools reported sensitivity ranges of 63%–90% and 62%–95% across multiple sites for detecting pneumothorax and pleural effusion, respectively.^4^ There are several proposed methods for improving generalizability, such as increasing the training sample size and diversity or federated model training.^5^^,^^6^ The former requires combining data from various institutions, but this can be difficult due to concerns about patient privacy.

Synthetic data generation for X-ray imaging has been approached from two main perspectives: physical simulation and statistical modeling. Physical simulation techniques, such as those described by Unberath et al., aim to model the underlying physics of X-ray image formation to generate realistic synthetic images.^7^ On the other hand, statistical modeling approaches utilise generative models to learn the data distribution and synthesise new samples. Image generation models face a trilemma to be an ideal solution—they must produce high-quality images with high diversity in a short period of time—excelling in all three areas simultaneously. Generative adversarial networks (GANs) have been widely used for this purpose as they can generate high quality images.^8^^,^^9^ GANs, on the other hand, suffer from *mode collapse*, where they cannot generate diverse images even as input prompts change.^10^ More recently, denoising diffusion probabilistic models (DDPMs) have emerged as a promising alternative to GANs, offering improved image diversity and quality. DDPMs can also be conditioned on medically relevant characteristics, creating images with specific attributes.^11^, ^12^, ^13^, ^14^ Chambon et al. and Packhauser et al. applied DDPMs to generate chest X-rays, showcasing their potential for synthetic data generation in this domain.^15^^,^^16^ However, as DDPMs denoise an image iteratively, the inference speed can be slow.

With advances in image generation models, there is hope that synthetic data can be used to address some of the aforementioned challenges with model performance and generalizability. Theoretically, by generating high-fidelity synthetic images as a means for dataset augmentation, we can *inject* some distribution characteristics into the training set, improving overall model performance.^17^ On the other hand, there are some concerns about performance degradation when using synthetic data iteratively, as it may lead to catastrophic interference, or in simpler words, model forgetting.^18^^,^^19^ The evidence regarding the use of synthetic data, either alone or supplementing real data, is controversial. For example, Packhauser et al. used diffusion generated chest radiographs for detecting chest pathologies and concluded that the classifier trained on synthetic images had lower performance compared to the one trained on real images.^15^ On the other hand, other studies have used synthetic data to show a model's performance will improve when we add model-generated versions of images to their real counterparts.^8^^,^^20^^,^^21^ However, these studies did not clearly delineate the underlying causes for improved performance, such as possible leakage of distribution characteristics, increased dataset size, increased variability, or disentangled distribution.

The goal of this study is to investigate the effect of synthetic data augmentation in medical imaging research. We first train a conditional DDPM on a subset of the CheXpert dataset and explore the optimal hyperparameters for dataset supplementation. Then we create a synthetic replica of this dataset that is up to 10 times larger than the source dataset by creating images with the same demographic and pathologic characteristics as the original dataset. By training several pathology classifiers using a mix of real and synthetic data and testing their performance on one internal and two external sources, we show the potential and limitations of synthetic data and investigate its failure modes.

## Methods

### Dataset description

For a comprehensive evaluation of merits of synthetic data in training data expansion, we collected all available frontal chest radiographs from the CheXpert (CXP), MIMIC-CXR (MIMIC) and Emory Chest X-ray (ECXR) datasets.^22^, ^23^, ^24^ All three datasets were annotated using the same automatic natural language processing (NLP) algorithm, CheXpert Labeler.^22^ CheXpert Labeler categorises 14 medical conditions into one of four categories based on the radiology reports: ‘Present’, ‘Absent’, ‘Not Mentioned’, and ‘Uncertain’. For the purpose of this study, we treated conditions that were ‘Not Mentioned’ in the reports as being ‘Absent’ or negative. When training DL models (DDPMs and classifiers), we excluded images that had *any* ‘Uncertain’ labels. However, for the testing phase, we included radiographs that had labels marked as ‘Uncertain’, but we omitted these conditions from our performance metric calculations. The preprocessing pipeline for all images included resizing them to 256 × 256 pixels while preserving the aspect ratio by padding and equalizing the image histogram to 256 bins. We used 4 A100 graphical processing units (GPUs) from NVIDIA (Santa Clara, CA, USA) for all generation and classification experiments. Splitting into train, tuning and test sets was done at the patient level for all three datasets.

CXP consists of 161,590 anteroposterior (AP) radiographs from 53,359 unique patients. For our training set (CXP_Tr_), we used 72,053 radiographs from 29,517 individuals who had complete demographic information. Radiographs with ‘Uncertain’ findings were excluded. For internal testing, we used *all* images (n = 56,448) from the remaining patients (CXP_Ts_). It has been previously shown that deep learning models can have different performances on AP and posteroanterior (PA) images, so we only used PA images and studied the effect of synthetic data supplementation on a dataset of only PA radiographs (MIMIC_PA_; see next).^4^

MIMIC-CXR contains 203,456 frontal radiographs from 68,412 distinct patients. We selected 184,587 radiographs from 58,898 individuals as the training set (MIMIC_Tr_). Consistent with CXP curation, radiographs with any ‘Uncertain’ findings were excluded. For testing (MIMIC_Ts_), all the images (n = 5961) from the remaining patients were used. Although MIMIC_Ts_ is small relative to the other sets, it only served for unbiased model evaluation in one of the experiments (see next). Additionally, we used two splits of the MIMIC dataset, namely MIMIC_AP_ (147,169 AP radiographs from 33,501 patients) and MIMIC_PA_ (96,155 PA radiographs from 45,628 patients), to study the effect of synthetic PA radiographs on AP and PA images from external sources. All dataset splits can be found in our GitHub repository (https://github.com/BardiaKh/SyntheticallyEnhanced).

Finally, to allow comparison of performance across models, 270,384 frontal radiographs (both AP and PA) from 162,113 patients from the ECXR dataset served as a common test set for all models. The dataset characteristics are summarised in Table 1 and Table E2.

**Table 1:** Study population characteristics. Pathology labels extracted using CheXpert labeler from the radiology reports are presented at an image level.

Variable	CXP_Tr_	CXP_Ts_	MIMIC^a^	ECXR
*Dataset statistics*				
Dataset origin	CA, USA	CA, USA	MA, USA	GA, USA
Number of images	72,053	56,448	243,324	270,384
Number of patients	29,517	23,842	63,945	162,113
*Demographic information*				
Age (IQR, yrs.)	51–75	47–74	52–76	41–67
Sex (female)	13,583 (46.02%)	10,847 (45.50%)	31,610 (49.43%)	87,777 (54.15%)
*Pathology labels*				
No finding	*pos:* 8167 (11.33%)	*pos:* 3286 (5.82%)	*pos:* 81,117 (33.34%)	*pos:* 127,100 (47.01%)
Enlarged cardiomediastinum	*pos:* 3775 (5.24%)	*pos:* 2638 (4.67%) *unc:* 3568 (6.32%)	*pos:* 7657 (3.15%) *unc:* 10,001 (4.11%)	*pos:* 15,475 (5.72%)
Cardiomegaly	*pos:* 10,080 (13.99%)	*pos:* 6456 (11.44%) *unc:* 2656 (4.71%)	*pos:* 47,673 (19.59%) *unc:* 6417 (2.64%)	*pos:* 49,396 (18.27%)
Lung lesion	*pos:* 2356 (3.27%)	*pos:* 1745 (3.09%) *unc:* 400 (0.71%)	*pos:* 6632 (2.73%) *unc:* 1192 (0.49%)	*pos:* 11,191 (4.14%)
Lung opacity	*pos:* 32,351 (44.90%)	*pos:* 30,119 (53.36%) *unc:* 1624 (2.88%)	*pos:* 54,769 (22.51%) *unc:* 4023 (1.65%)	*pos:* 37,380 (13.82%)
Edema	*pos:* 23,430 (32.52%)	*pos:* 15,893 (28.16%) *unc:* 5296 (9.38%)	*pos:* 29,331 (12.05%) *Unc:*14,244 (5.85%)	*pos:* 15,287 (5.65%)
Consolidation	*pos:* 4940 (6.86%)	*pos:* 3943 (6.99%) *unc:* 9967 (17.66%)	*pos:* 11,525 (4.74%) *unc:* 4598 (1.89%)	*pos:* 6331 (2.34%)
Pneumonia	*pos:* 1599 (2.22%)	*pos:* 1195 (2.12%) *unc:* 6584 (11.66%)	*Pos:*17,222 (7.08%) *unc:* 19,441 (7.99%)	*pos:* 7303 (2.70%)
Atelectasis	*pos:* 13,518 (18.76%)	*pos:* 9130 (16.17%) *unc:* 12,526 (22.19%)	*pos:* 48,790 (20.05%) *unc:* 10,965 (4.51%)	*pos:* 30,815 (11.40%)
Pneumothorax	*pos:* 9171 (12.73%)	*pos:* 4381 (7.76%) *unc:* 953 (1.69%)	*pos:* 11,235 (4.62%) *unc:* 1205 (0.50%)	*pos:* 8631 (3.19%)
Pleural effusion	*pos:* 32,872 (45.62%)	*pos:* 21,856 (38.72%) *unc:* 3702 (6.56%)	*pos:* 57,721 (23.72%) *unc:* 6202 (2.55%)	*pos:* 30,205 (11.17%)
Pleural other	*pos:* 654 (0.91%)	*pos:* 488 (0.86%) *unc:* 542 (0.96%)	*pos:* 2083 (0.86%) *unc:* 794 (0.33%)	*pos:* 4861 (1.80%)
Fracture	*pos:* 2892 (4.01%)	*pos:* 2085 (3.69%) *unc:* 253 (0.45%)	*pos:* 4781 (1.96%) *unc:* 602 (0.25%)	*pos:* 4826 (1.78%)
Support devices	*pos:* 45,032 (62.50%)	*pos:* 33,127 (58.69%) *unc:* 360 (0.64%)	*pos:* 73,294 (30.12%) *unc:* 267 (0.11%)	*pos:* 119,232 (44.10%)

Abbreviations: CXP_Tr_, CheXpert Train; CXP_Ts_, CheXpert Test; ECXR, Emory Chest X-ray; pos, Positive finding by Labeler; unc: Uncertain Finding by Labeler.

a Demographic information is only available for 60,523 patients.

### Image generation

**Fig. 1:**
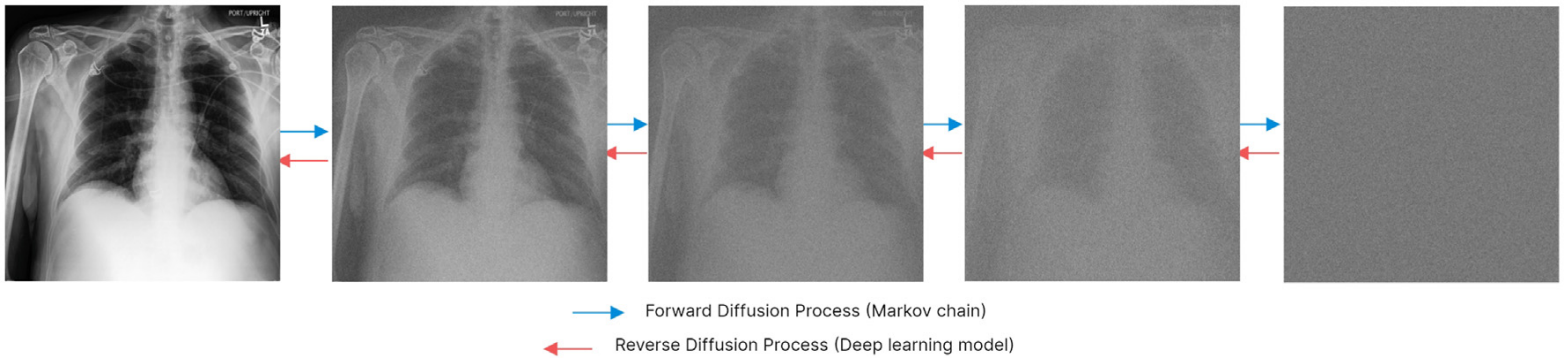
Overview of forward and reverse diffusion processes.

We used denoising diffusion probabilistic models (DDPMs) to create synthetic images.^25^ DDPMs work by combining forward and reverse diffusion processes, Fig. 1. Additional details about the diffusion-based image generation can be found in our Supplementary Materials. We employed the Mediffusion package (v0.6.0) to train a generative model based on the CXP_Tr_ set.^13^ We conditioned the model on sex, age, race, and the 14 pathology labels. Demographic information was obtained from self-reported patient records. The model was trained for 1.1 million steps. A detailed model configuration can be found in our repository and Table E1. After training, we sampled pure Gaussian noise and denoised it gradually by passing it iteratively through the diffusion model. For faster sampling during inference, we employed implicit sampling with 200 denoising steps.^26^

To make the generated images correspond to the conditioning variables, we used classifier-free guidance (CFG). To investigate the impact of the CFG scale on downstream tasks, we made three replicas of the CXP_Tr_ with the exact same demographic and pathology labels. These synthetic replicas were differentiated by CFG scales set at {0, 4, 7.5}. We set aside 10% of the CXP_Tr_ for tuning and used the remaining 90% to train a classifier to predict the 14 labels. Through 10 experiments, where we independently substituted the training and tuning sets with their synthetic counterparts, and assessed the influence of the CFG scale on classification tasks. The hyperparameters of the pathology classifier are discussed in the next section.

After identifying the most appropriate CFG scale, we generated a large synthetic dataset. In this dataset, every real image in CXP_Tr_ was replicated into 10 synthetic variants. Each variant retained the same demographic and pathology attributes but was created with different initialization seeds to add another level of diversity to the synthetic dataset.

### Pathology classification

We devised a series of classification tasks to predict 14 condition labels from chest radiographs. A ConvNeXt-base model pretrained on natural image datasets was used for all experiments. An input size of 256 × 256 pixels with standard set of augmentations was used, hyperparameters are presented in Table E1. To further stabilise the training, we used exponential moving weight averaging (EMA) with a decay factor of 0.9999.^27^ We trained all models for a maximum of 50 epochs, and the best model was selected based on the lowest loss value on the tuning set. This ensured a fair comparison between the baseline and synthetically supplemented models.

In order to systematically evaluate the effect of synthetic data on downstream tasks, we defined three sets of experiments. In all experiments, the models had the same hyperparameters, and the only varying factor was the input data. Details and the rationale behind each experiment are as follows.

#### Supplementing real data with synthetic data from the same origin

The purpose of this experiment was to assess if synthetic data from a dataset could increase model performance on the same test set distribution. To this end, we used a graded regimen of synthetic data (100%–1000% in 100% increments) and added it to the real training set of our classifier. As an example, 300% supplementation means that we added three times as many synthetically generated sets of images to the original images and used them for model training. We randomly selected 10% of the CXP_Tr_ for tuning, and the rest were used for model training. Of note, we only used synthetic images for expanding training and did not include synthetic images in the tuning set. To have a baseline comparison, we trained a model using only real data (0% supplementation ratio).

#### Purely synthetic data

This experiment is designed to gauge the performance of a model trained only on synthetic data, simulating an instance where only synthetic data is shared with an outside institution. This experiment helps establish the *utility* of synthetic data alone and shows the extent to which synthetic data can replace real data without sacrificing performance. We used the same split as the previous experiment and excluded any real data from the training set, while keeping the tuning set real.

#### Mixing synthetic data with an external dataset

The objective of this experiment was to evaluate the generalizability of our model when trained on a combination of real and synthetic data from different distributions. We used MIMIC_Tr_ as the training set (split in 90%:10% for training and tuning) and mixed 10 different ratios of synthetic data (generated based on CXP_Tr_), similar to previous experiments.

### Evaluation

We used Fréchet Inception Distance (FID) to evaluate the quality and diversity of generated synthetic images compared with their real counterparts. To assess the performance of pathology classifiers, we used area under receiver operating curve (AUROC) as our main metric. To calculate the standard deviation of the models' performance, we bootstrapped the results 1000 times and compared them using independent t-test. Additionally, we adjusted the probability of committing a type I error (α) using the Bonferroni correction for multiple comparisons. An α = 0.05 was considered significant in all instances. Finally, to understand label distributions, we used a pathology co-occurrence matrix for each dataset and compared the similarity between them using Pearson's correlation coefficient. Inference speed measurements are reported based on an 80 GB A100 GPU.

### Ethics

Publicly available datasets were used to train image generation and disease classifier models. The Emory CXR dataset was curated after obtaining approval from the Emory University Institutional Review Board (#STUDY00000506), and the need for informed consent was waived due to minimal incurred risk for the patients. All procedures and methods were carried out in accordance with the guidelines and regulations set forth by the Institutional Review Board.

### Role of funders

The funding bodies did not influence the research strategy and content of the manuscript.

## Results

### Synthetic data quality

**Fig. 2:**
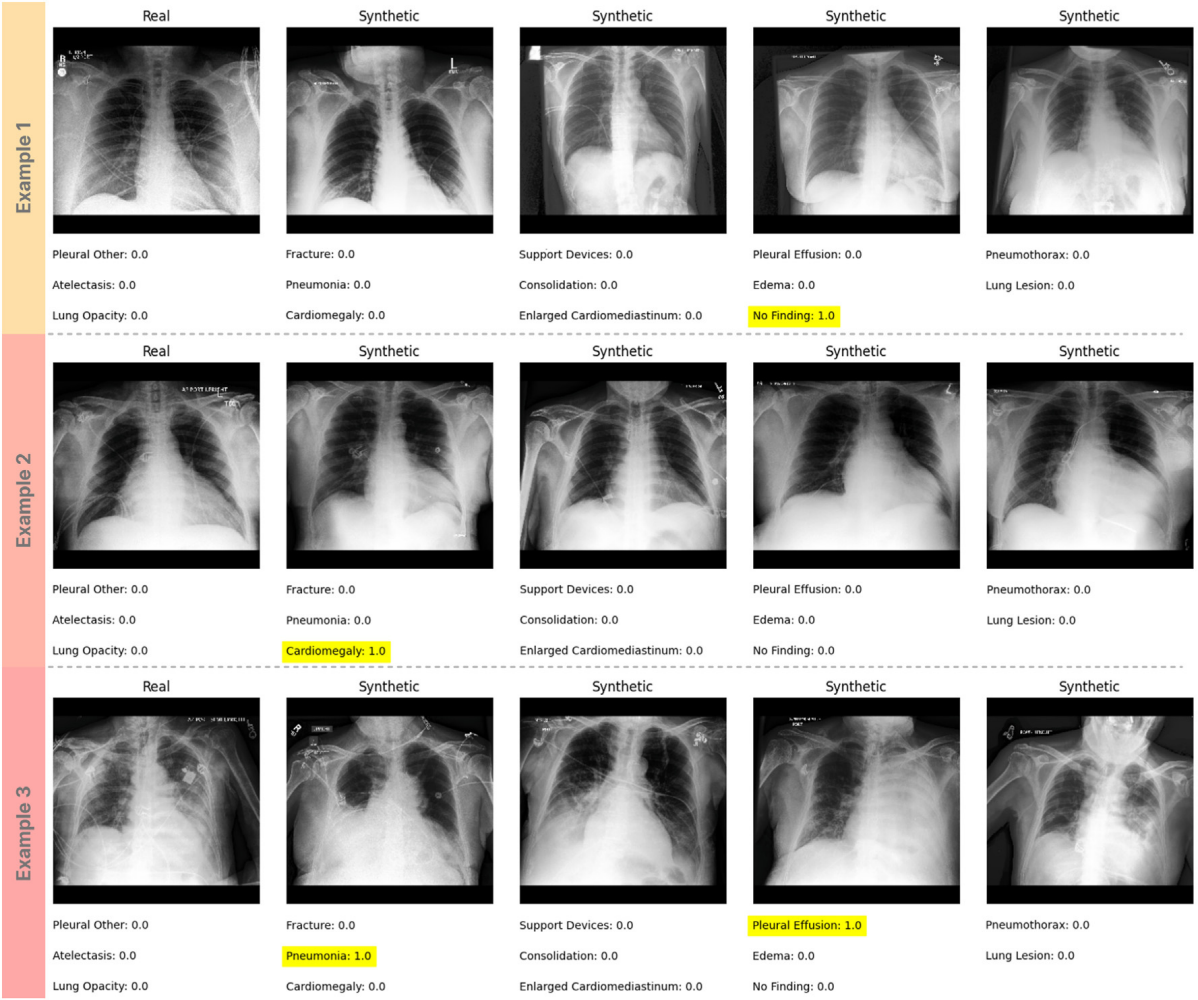
Examples of the real and synthetic images obtained from the diffusion model using different seeds. Presented pathologies are what the model was actually conditioned on.

Training the diffusion model took 336 A100 GPU hours. Fig. 2 represents some of the generated samples along with their conditioned pathology labels. During the initial round of inference, using three different classifier-free guidance (CFG) scales of 0, 4, and 7.5, the model had an FID of 6.4, 7.4, and 13.9, respectively. The inference time for creating a single image with CFG scales of 4 and 7.5 (13.65 s and 13.74 s, respectively; SD: 0.05) was twice the amount of time for generating images with a CFG scale = 0 (6.84 s, SD: 0.04). In terms of performance, the model trained on CFG scale = 0 had an AUROC of 0.797 on the tuning set, compared to the model trained on real images (AUROC = 0.805). Table 2 represents the findings of this set of experiments. Based on these results, we selected a CFG scale = 0 for creating the large synthetic 720 K image dataset.

**Table 2:** The effect of CFG scale of generated images on downstream classifier model performance.

	Tuning set			
	Real	CFG = 0	CFG = 4	CFG = 7.5
**Training set**				
Real	0.8053	0.7911	0.9199	0.9245
CFG = 0	0.7969	0.8016	–	–
CFG = 4	0.7406	–	0.9671	–
CFG = 7.5	0.6983	–	–	0.9839

Abbreviation: CFG, classifier-free guidance.

All numbers are presented as AUROCs

### Classification experiments

#### Supplementing real data with synthetic data from the same origin

**Fig. 3:**
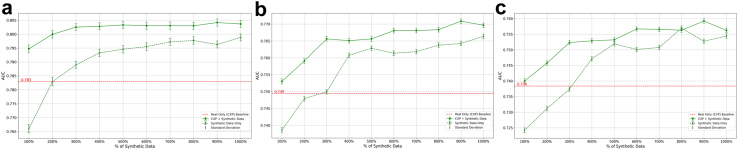
Performance evaluation of models trained on real data, real data supplemented by synthetic data, and synthetic data only on various test sets: (a) CheXpert Test, (b) MIMIC-CXR, and (c) Emory Chest X-ray. The red line in all graphs represents the baseline classifier model's (trained only on real data from the CheXpert training set; CXP_Tr_) performance on the target dataset. Error bars represent the standard deviation of the metric based on bootstrapping.

By incrementally adding synthetic data to real samples to the classifier training data, AUROC increased from a baseline of 0.782 (no synthetic data) to 0.804 (with 1000% data supplementation) on CXP_Ts_ (p-value <0.01). Upon external testing on MIMIC_all_ adding 1000% synthetic data supplementation resulted in increasing the model AUROC from 0.749 to 0.770 (p-value <0.01). By analyzing the AP and PA subgroups, we saw an increase in performance on both the MIMIC_AP_ and MIMIC_PA_ subsets (0.020 and 0.014, respectively, p-value <0.01). External testing on ECXR showed a 0.017 increase in performance, from 0.739 to 0.756, when maximal data supplementation occurred (p-value <0.01). Fig. 3 summarises these findings for all the intermediary supplementation steps.

#### Purely synthetic data

Our experiments showed similar trends when using only synthetic data. For example, on CXP_Ts_, training the classifier on 200% of synthetic data showed the same performance as training only on real data (AUCROC of 0.783 in both cases; p-value: 0.98). The model achieved similar performance on MIMIC_all_ and ECXR when using 300% synthetic data. However, all models trained on synthetic data alone performed worse than the comparable models trained on real and synthetic data. Fig. 3 compares the iterative supplementation of synthetic replicas and shows the effect of real data in the three datasets.

#### Mixing synthetic data with an external dataset

**Fig. 4:**
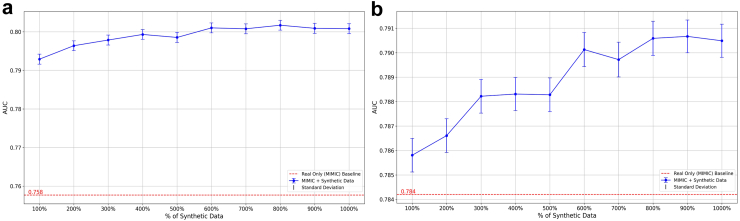
Performance evaluation of models trained on the MIMIC-CXR training set (MIMIC_Tr_) with and without supplementation with synthetic data from external sources on various datasets: (a) CheXpert Test, and (b) Emory Chest X-ray. Error bars represent the standard deviation of the metric based on bootstrapping.

When a classifier model was trained using data from two sites - MIMIC_Tr_ and 1000% supplementation of synthetic images from CXP_Tr_, the model performance increased from 0.790 to 0.796 on MIMIC_Ts_ (p-value <0.01). Additionally, the same model had a 0.007 AUROC increase when tested on ECXR (baseline: 0.784, 1000% supplementation: 0.791; p-value <0.01). With only 100% supplementation, the model performance on CXP_Ts_ increased from 0.758 to 0.793 (p-value <0.01). Adding 10 replicas of CXP_Tr_ to MIMIC_Tr_, resulted in a final AUROC of 0.801 (p-value <0.01 compared with 100% supplementation). Fig. 4 shows the effect of graded synthetic data supplementation on model performance on CXP_Ts_ and ECXR.

Tables E3–E9, show pathology-specific results for all models. These results suggest that the largest increase in performance was in pathologies with <5% prevalence in the population. Additionally, to further investigate the model performance gaps, we created co-occurrence matrices for the three datasets (Figures E1–E3). We found that MIMIC-CXR and ECXR had the highest correlation (0.862), followed by MIMIC-CXR and CXP (0.846). The least label correlation was between CXP and ECXR, which was 0.789.

## Discussion

Synthetic data has been used for many years as a way to tackle class imbalances in tabular data analysis.^28^ However, their use has been limited in medical imaging due to the low quality of the synthetic data. With the emergence of newer techniques, such as diffusion models, there is an opportunity to create high quality diverse medical images and see their effect on downstream task performance. Our study demonstrates that DL models trained on synthetic data can achieve performance levels comparable to those trained on real data, highlighting the viability of synthetic datasets in medical imaging. Additionally, supplementing real datasets with synthetic data significantly enhances model performance and generalizability, underscoring the potential of synthetic data in improving model robustness.

Our preliminary experiments showed that a classifier-free guidance (CFG) scale of 0 results in synthetic images that are most similar to real images. By increasing the CFG scale, we are *overexpressing* the conditioning signal, making the model trained on higher CFGs not learn the more nuanced pathology signals in real images, hence the lower performance. Additionally, a model trained on real images has a much easier task of detecting the *overexpressed* signal on synthetic tuning sets generated by CFG >0.

By supplementing real radiographs with synthetic replicas of the same origin (both from the CheXpert dataset), model performance increased, and the increase was incremental with addition of more synthetic images. We observed a 0.020 increase in average AUROC in internal and external test sets. Interestingly, although the synthetic images were all AP, the performance boost was seen with *both* AP and PA images. This suggests increased dataset diversity is one of the factors contributing to better performance. Additionally, by using synthetic data alone, we found that by using 200–300% of synthetic data, we can have a model that matches the performance of a model trained on real data. However, the model performance lags behind the model that was trained with real and synthetic data, highlighting the value of real images. This verifies previous reports that synthetic data alone will not match real data performance.^15^ Furthermore, the performance gap on the Emory dataset between models trained on MIMIC and models trained on CheXpert with synthetic data supplementation highlights the limitations of synthetic data in capturing the characteristics of datasets outside its training range. As our results show, CheXpert and Emory datasets have the lowest label correlation among the three datasets studied, which may contribute to the observed performance differences.

Finally, by mixing synthetic data with real data from another institution (synthetic CXP_Tr_ and MIMIC_Tr_) we observe that the model generalises better (increased AUROC of 0.043) on the source distribution (CXP_Ts_). However, the increased performance on the CXP tuning set was more pronounced (0.070 AUROC increase) than CXP_Ts_. This suggests that there is information leakage in synthetic data, raising methodological concerns for previous studies in which the test set for evaluating the performance of synthetic data was not explicitly separate from the generative model training or tuning source.^16^^,^^29^^,^^30^ We believe that to appropriately measure performance gains from synthetic data, the test set must be from a different distribution than the source of the synthetic data; otherwise, performance measurements may be inflated.

Our image synthesis pipeline demonstrates superior performance compared to other generative models in the literature. GAN-based models have been shown to suffer from mode collapse, as evidenced by the high Fréchet Inception Distance (FID) score of 148 reported by Han et al. for their high-resolution GAN-based image generator.^8^ In contrast, diffusion-based models have shown more promising results. Chambon et al. reported an FID of 55 for their best-performing latent diffusion model, while Weber et al. achieved an FID of 12 using a cascaded diffusion pipeline.^16^^,^^31^ Although FID can be resolution-dependent, a direct comparison with the work of Pan et al., who trained a diffusion model on 256 × 256 pixel radiographs, highlights the superiority of our approach. Our model achieves an FID of 6.4, substantially outperforming their reported FID of 22.3.^32^ A notable challenge with diffusion models is their slow inference time, necessitated by the iterative denoising process of image generation. Consequently, a single replication of the training set (72,000 images) demands approximately 137 A100 GPU-hours, which can become expensive depending on the compute provider. The decision to balance enhanced performance derived from synthetic data supplementation against associated inference costs should be made on a case-by-case basis, taking into consideration specific requirements and constraints of each research project.

One important implication of using synthetic data is privacy concerns. Several studies have pointed out the possibility of data leaks in generative models. Specifically, it has been shown that if there are multiple copies of a person's face with the same prompt in a dataset, a diffusion model can associate the face with the person's name and leak training data.^33^ This can be concerning in the setting of healthcare, where patient anonymity is of utmost importance. There have been some solutions proposed to ensure training data is not generated, or at least not released, but these are all experimental.^34^ Another short term solution would be scanning all generated images using PHI redaction models, but this is just a temporary solution.^35^ As the research frontier tackles synthetic data anonymization, we can only experiment with hypothetical scenarios from publicly available datasets.

Our results should be interpreted with attention to some limitations. First, we used labels extracted by the CheXpert labeler as our conditioning variable for image generation. Since this tool relies on rule-based techniques, there might be abstraction errors that can influence the image quality and ground truth labels for classification.^36^ Second, we only used images with one CFG scale. Using a stepwise increase in the CFG scale to create different datasets might help with training a model on both easy and hard cases. Third, we only investigated the effect of synthetic data supplementation on downstream classification tasks; similar rigorous validation is required for other tasks, such as segmentation and object detection. Fourth, due to the extent of experiments with 10× data supplementation and our computational constraints, we selected 256 as our generated image size, which is similar to previous works.^37^, ^38^, ^39^ A cascaded diffusion pipeline can be further used to increase the size of the generated images to higher resolutions.^40^ Additionally, the diffusion model can be nested in a larger model to train a model that directly outputs high resolution images.^41^ Finally, we only replicated the dataset without changing the disease prevalence to show only the potential of synthetic data without disentangling disease distribution. In future studies, oversampling of specific pathologies with the lowest prevalence should be investigated. It is also important to test the limit of generative model capabilities in generation of these low-prevalence conditions for long-tailed disease classification.^42^

In conclusion, we showed that synthetic data can be useful for training downstream classifier models and that, in large numbers, they can match or outperform the classifiers trained on real data. We further showed the optimal hyperparameters for generating synthetic datasets and showed that the findings are generalizable in two large datasets with more than 500,000 radiographs. Importantly, even a modest amount of synthetic data can close the generalization gap of models trained on other data sources. Finally, we show that real data quality is still superior to synthetic data, and gathering more data should be the first solution for increasing dataset size.

## Contributors

Conceptualization: B.K., F.L., B.J.E. and J.W.G.; Data curation: B.K., F.L., T.D., P.R. and C.U.G.; Formal analysis: B.K., F.L., T.D., P.R. and C.U.G.; Funding acquisition: B.J.E. and J.W.G.; Investigation: B.K., F.L., T.D., B.J.E. and J.W.G.; Methodology: B.K., F.L., T.D., P.R., C.U.G., H.M.T., C.C.W., A.B.S., S.P., B.J.E. and J.W.G.; Project administration: B.K., F.L., B.J.E. and J.W.G.; Resources: B.K., F.L., T.D., P.R., C.U.G., H.M.T., C.C.W., S.P., B.J.E. and J.W.G.; Software: B.K., F.L. and P.R.; Supervision: H.M.T., C.C.W., A.B.S., S.P., B.J.E. and J.W.G.; Validation: B.J.E. and J.W.G.; Visualization: B.K., F.L. and T.D.; Writing—original draft: B.K., F.L., B.J.E. and J.W.G.; Writing–review & editing: B.K., F.L., T.D., P.R., C.U.G., H.M.T., C.C.W., A.B.S., S.P., B.J.E. and J.W.G.; All authors have read and approved the final version of the manuscript.

## Data sharing statement

The CheXpert dataset and MIMIC-CXR are all publicly available. The Emory CXR is available on request after signing a data use agreement. All dataset splits and codes, including training and validation of image generation and pathology classification models, are available at https://github.com/BardiaKh/SyntheticallyEnhanced. DDPM checkpoints are also available on the same GitHub repository. Classifier checkpoints will be shared upon request from the authors.

## Declaration of interests

A.B.S. has stocks and options in Google, LLC. B.K., P.R., C.C.W., and B.J.E. have pending patents on radiographic image generation and feature extraction from generative models (63/583,044 and PCT/US2023/074,166). J.W.G. is a member of the American College of Radiology (ACR) AI advisory group, Society of Imaging Informatics in Medicine (SIIM) board, and the Health Level 7 (HL7) Standards board.

## Appendix A Supplementary data

Supplemental Figures

Supplemental Methods Explanation

Supplemental Methods Tables

Supplemental Results Tables

Supplemental Results Tables
