# Supplementary material for: Synthetically enhanced: unveiling synthetic data's potential in medical imaging research
Source: eBioMedicine. 2024 May 30;104:105174. doi: 10.1016/j.ebiom.2024.105174 (PMC11177083; doi:10.1016/j.ebiom.2024.105174)

**Figure E1**. Normalized label co-occurrence matrix for pathologies in the CheXpert dataset. For each condition on the row (*r*) of the heatmap, the corresponding column (*c*) indicates the ratio of all samples with condition *r* that also have condition *c*.


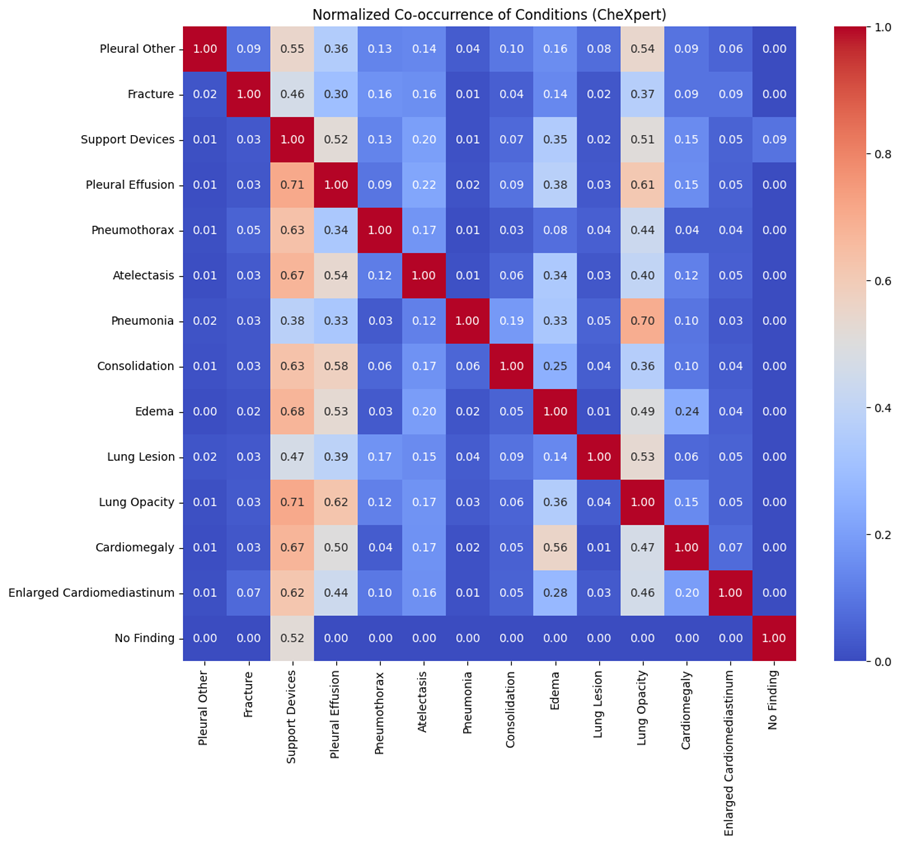


**Figure E2**. Normalized label co-occurrence matrix for pathologies in the MIMIC-CXR dataset. For each condition on the row (*r*) of the heatmap, the corresponding column (*c*) indicates the ratio of all samples with condition *r* that also have condition *c*.


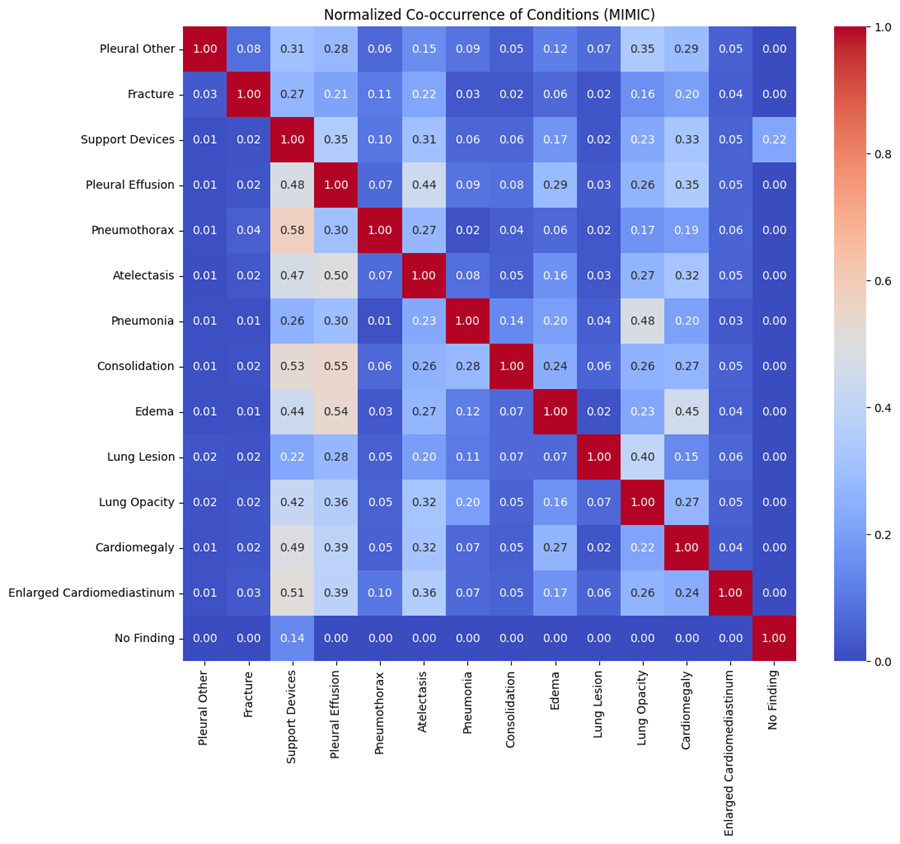


**Figure E3**. Normalized label co-occurrence matrix for pathologies in the Emory Chest X-ray dataset. For each condition on the row (*r*) of the heatmap, the corresponding column (*c*) indicates the ratio of all samples with condition *r* that also have condition *c*.


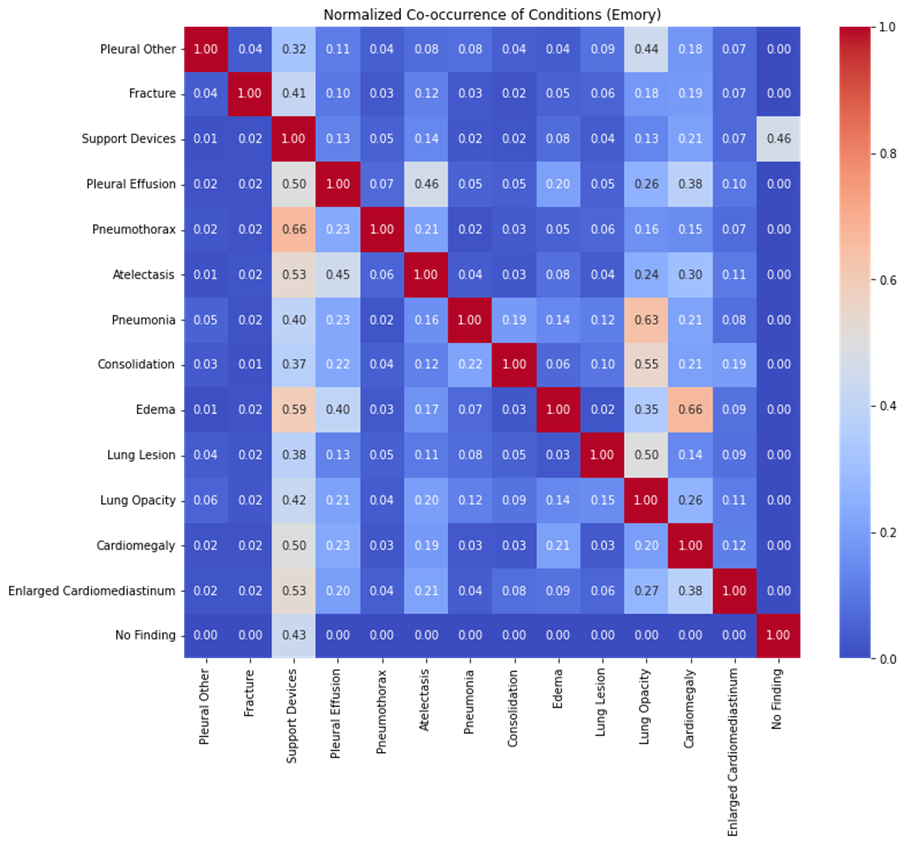

Supplement: Supplemental Figures [file mmc1.docx]
