# Supplementary material for: Synthetically enhanced: unveiling synthetic data's potential in medical imaging research
Source: eBioMedicine. 2024 May 30;104:105174. doi: 10.1016/j.ebiom.2024.105174 (PMC11177083; doi:10.1016/j.ebiom.2024.105174)
Supplement: Supplemental Methods Explanation [file mmc2.docx]

## Supplementary Materials

DDPMs work by combining forward and reverse diffusion processes. The stepwise incorporation of small amounts of Gaussian noise into a starting image is termed the forward diffusion process. With an increasing number of steps, denoted by total timesteps ($T$), the initial image will gradually morph into isotropic Gaussian noise. This sequence is characterised by a Markovian function, which is defined by a noise schedule ($\beta$). This implies that to reach the noisy image at timestep 150 ($t=150$), one must sequentially pass through the first 149 timesteps. Nonetheless, due to the characteristics of Gaussian-distributed noise, it is possible to deduce the appearance of an image at step 150 without having to add noise to it in the preceding 149 steps. Using the base image ($x_{0}$), we can determine its noisier counterpart at any chosen timestep ($x_{t}$) with the equation:

$x_{t}=\sqrt{\underline{\alpha}_{t}} x_{0} +\sqrt{{1-\underline{\alpha}}_{t}} \epsilon$

where:

$\alpha_{t}=1-\beta_{t}$ ; $\underline{\alpha}_{t}=\prod_{s=0}^{t} \alpha_{s}$

The noise schedule ($\beta$) is set in advance of the training, which then establishes the values of $\alpha_{t}$and $\underline{\alpha}_{t}$ (cumulative product from $\alpha_{0}$ to $\alpha_{t}$ for each timestep $t$). In each training step, $\epsilon$ (from Gaussian distribution) is sampled for each entry in the batch. This noise is paired with the aforementioned equation to produce $x_{t}$. The reverse diffusion procedure aims to estimate the noise addition ($\epsilon$) between consecutive steps. Contrary to the more direct forward diffusion, achieving reverse diffusion is more demanding and involves training a deep learning (DL) model, often referred to as a diffusion model. The main goal during training is to reduce the mean squared error (MSE) loss between the noise as predicted by the diffusion model and the actual original noise ($\epsilon$), which is precomputed through the forward diffusion technique.

To make the generated images correspond to the conditioning variables, we used classifier-free guidance (CFG). Unlike other techniques, CFG does not require the training of a separate classifier to condition the diffusion model. It operates by utilizing a learned *null embedding* that is randomly swapped with actual class embeddings during training. During inference, the CFG scale dictates how closely the generated image corresponds to the conditioning variables. As with CFG scales > 0, two model passes are required to create an image, we anticipate faster sampling with CFG scale =0.
