# Supplementary material for: Synthetically enhanced: unveiling synthetic data's potential in medical imaging research
Source: eBioMedicine. 2024 May 30;104:105174. doi: 10.1016/j.ebiom.2024.105174 (PMC11177083; doi:10.1016/j.ebiom.2024.105174)
Supplement: Supplemental Methods Tables [file mmc3.docx]

**Table E1.** Model hyperparameters.

| **Hyperparameter** | **Value** |
| --- | --- |
| *Diffusion Model* | |
| Input Shape | 256 x 256 pixels |
| Noise Schedule | Cosine |
| Total Timesteps (T) | 1000 |
| Attention Resolution | 32, 16, 8 |
| UNet Channels | 128 |
| UNet Channel Multipliers | 1, 1, 2, 2, 4, 4 |
| Number of Residual Blocks | 2 |
| Number of Attention Heads | 4 |
| Number of Conditioning Classes | 22 in total:   - 14 pathologies [extracted by CheXpert Labeler] - 1 age [in decades, e.g., 67 was encoded as 6] - 2 sexes [Male / Female] - 5 races [African American / American Indian / Asian / Pacific Islander / White]) |
| Guidance Drop Rate (During Training Only) | 0.1 |
| *Classifier Models* | |
| Architecture | ConvNeXt-base |
| Pretraining Dataset | ImageNet |
| Input Size | 256 x 256 pixels |
| Online Augmentations | - Horizontal and vertical flipping  - Rotation (± 60 degrees)  - Resizing (± 10%)  - Translation (± 12 pixels) |
| Learning Rate | 0.00001 |
| Weight Decay | 0.0003 |
| Optimizer | Lion |
| Loss Function | Binary cross-entropy |
| Exponential Moving Weight Averaging (EMA) Decay Factor | 0.9999 |

**Table E2**. Population characteristics for MIMIC-CXR subsets. Pathology labels extracted using CheXpert labeler from the radiology reports are presented at an image level.

Abbreviations: MIMIC_Tr_, MIMIC Train; MIMIC_Ts_, MIMIIC Test; pos, Positive finding by Labeler; unc: Uncertain finding by Labeler.

‡ Demographic information is only available for 55,737 patients.

✝Demographic information is only available for 4,266 patients.

| **Variable** | **MIMIC_Tr_**^‡^ | **MIMIC_Ts_**^✝^ |
| --- | --- | --- |
| *Dataset Statistics* | | |
| Dataset Origin | MA, USA | MA, USA |
| Number of Images | 184,587 | 5,961 |
| Number of Patients | 58,898 | 4,503 |
| *Demographic Information* | | |
| Age (IQR, yrs.) | 51-75 | 47-74 |
| Sex (female) | 29,222  (49.61%) | 2,124  (47.17%) |
| *Pathology Labels* | | |
| No Finding | *pos:* 5163  (2.80%) | *pos:* 180  (3.02%)  *unc:* 447  (7.50%) |
| Enlarged Cardiomediastinum | *pos:* 34570  (18.73%) | *pos:* 922  (15.47%)  *unc:* 380  (6.37%) |
| Cardiomegaly | *pos:* 4662  (2.53%) | *pos:* 225  (3.77%)  *unc:* 224  (3.76%) |
| Lung Lesion | *pos:* 27946  (15.14%) | *pos:* 2871  (48.16%)  *unc:* 453  (7.60%) |
| Lung Opacity | *pos:* 20228  (10.96%) | *pos:* 501  (8.40%)  *unc:* 1215  (20.38%) |
| Edema | *pos:* 5903  (3.20%) | *pos:* 351  (5.89%)  *unc:* 375  (6.29%) |
| Consolidation | *pos:* 11723  (6.35%) | *pos:* 483  (8.10%)  *unc:* 2554  (42.85%) |
| Pneumonia | *pos:* 34175  (18.51%) | *pos:* 1027  (17.23%)  *unc:* 1419  (23.80%) |
| Atelectasis | *pos:* 9007  (4.88%) | *pos:* 44  (0.74%)  *unc:* 77  (1.29%) |
| Pneumothorax | *pos:* 38390  (20.80%) | *pos:* 906  (15.20%)  *unc:* 671  (11.26%) |
| Pleural Effusion | *pos:* 1394  (0.76%) | *pos:* 67  (1.12%)  *unc:* 122  (2.05%) |
| Pleural Other | *pos:* 3662  (1.98%) | *pos:* 134  (2.25%)  *unc:* 257  (4.31%) |
| Fracture | *pos:* 52022  (28.18%) | *pos:* 717  (12.03%)  *unc:* 33  (0.55%) |
| Support Devices | *pos:* 5163  (2.80%) | *pos:* 180  (3.02%)  *unc:* 447  (7.50%) |
